# The Antitumor Potential of Extract of the Oak Bracket Medicinal Mushroom *Inonotus baumii* in SMMC-7721 Tumor Cells

**DOI:** 10.1155/2019/1242784

**Published:** 2019-09-22

**Authors:** Yue Yang, Pingya He, Ning Li

**Affiliations:** Anhui Key Laboratory of Bioactivity of Natural Products, School of Pharmacy, Anhui Medical University, Hefei 230032, China

## Abstract

*Inonotus baumii*, a traditional medicinal mushroom, has been historically used in China and other countries of East Asia for the treatment of various diseases. The aim of this study is to investigate the antitumor activity of the extract of *I. baumii* (EIB) against hepatocellular carcinoma and the possible mechanism involved. The MTT assay was used to evaluate the proliferative activity of SMMC-7721 cells treated with EIB. Hoechst 33258 and JC-1 staining were used to determine nuclear morphological changes and mitochondrial membrane potential, respectively. Flow cytometry analysis indicated that EIB blocked the cell cycle at the S phase and induced significant apoptosis. EIB increased the protein expression of Bax, cytochrome c, cleaved caspase-3, and decreased Bcl-2 in SMMC-7721. Moreover, EIB induced autophagy, indicated by the increase of autophagy-related protein expression of LC3-II and decrease of p62, and the AMPK/mTOR/ULK1 pathway was involved in the autophagic cell death. *In vivo*, EIB was found to strongly inhibit the growth of tumors in BALB/c nude mice. Our results indicated that *I. baumii* might be a potential natural therapeutic agent for liver cancer, as it could induce apoptosis and autophagy in HCC cells.

## 1. Introduction

Hepatocellular carcinoma (HCC) is the most common primary liver malignancy [1]. The incidence of HCC is highest in Asia and sub-Saharan Africa, and China accounts for about 55% of the global HCC cases [2, 3]. Despite the progress in surgery, radiotherapy, and chemotherapy or liver transplantation, the current five-year overall survival rate of HCC is only 30–40% [4]. The available therapeutic options are still limited due to various adverse effects, as well as the high incidence of cancer recurrence and metastasis, which may lead to poor prognosis for HCC patients. For early-stage HCC patients, less than 30% of them can be treated with surgical resection, orthotopic liver transplantation, and percutaneous ablation [5]. For patients with advanced-stage HCC, the abovementioned treatments are no longer available, and systemic treatment is indicated [6], where sorafenib and lenvatinib are the systemic agents [7]. However, there is no single treatment to achieve the desired effect, and new treatments are urgently needed. Nowadays, natural herb medicines in HCC treatment are gaining more attention because of their advantages of efficiency, safety, and few side effects [8].

Cell death is ubiquitous for all organisms. Programmed cell death (PCD) is the death of a cell mediated by an intracellular program. PCD plays a critical role in the survival/death balance in metazoan cells, and the regulation of PCD is an important target in cancer chemotherapy [9]. PCD can be divided as two forms: apoptosis (type I) and autophagy (type II). Apoptosis is a caspase-mediated PCD and characterized by chromosome condensation, nuclear fragmentation, membrane blebbing, and the formation of apoptotic bodies [10]. In contrast to apoptosis, autophagy is a catalytic process, accompanied by the formation of the autophagosome in which excess protein or subcellular components are degraded by lysosomes [11]. Autophagy acts as a prosurvival mechanism but can also induce autophagic cell death.

*Inonotus baumii* (Pilát) T. Wagner & M. Fisch, a kind of medicinal mushroom, hosted on the broad-leaved trees such as poplar and willow, is mainly distributed in China, Korea, and some other Asian countries [12]. *I. baumii* has been historically used as a health supplement and herbal medicine in East Asian areas [13]. It has been reported that *I. baumii* has various biological activities including improving immunity [14] and anticancer [12], anti-inflammatory [12], and antibacterial [15] activity. At present, much work has not been focused on investigating the antitumor mechanism of *I. baumii*.

Previously, we reported the antitumor effects of the different extracts of *Phellinus igniarius*, a willow bracket medicinal mushroom, in MGC-803 [16] and SGC-7901 cells [17]. In fungus-host interactions, different hosts produce significant metabolites and secondary metabolites for parasites, resulting in different biological activities. In this study, we evaluated the antitumor effects of *I. baumii*, an oak bracket medicinal mushroom, against SMMC-7721 cells through two signaling pathways. Initially, through the cytotoxicity assay *in vitro*, we found that the extract of *I. baumii* had obvious cytotoxic effects on SMMC-7721 cells. In order to provide other scientific proofs, particularly to support the mechanisms of the healthcare benefits of this medicinal mushroom, this study focused on the antitumor effects of *I. baumii* on SMMC-7721 and relevant molecular mechanisms.

## 2. Materials and Methods

### 2.1. Chemicals and Reagents

The Hoechst staining kit and mitochondrial membrane potential assay kit with JC-1 were purchased from Beyotime. MTT was purchased from Biofrox. Antibodies of AMPK, p-AMPK, mTOR, p-mTOR, ULK1, p-ULK1, p62, Beclin-1, and *β*-actin were purchased from Cell Signaling Technology. Antibodies of cleaved caspase-3, Bax, Bcl-2, and LC3 were purchased from Abcam.

### 2.2. Mushroom Materials and Preparation of Extract

The fruiting bodies of *I. baumii* were provided by Jinzhai Shangzhen Biological Co., Ltd., Anhui Province, China, in November 2018, and were identified by Prof. Kai-Jin Wang from Anhui University, where a voucher specimen (no. 20181101) was deposited. The dried fruiting bodies of *I. baumii* were cut into small pieces and extracted three times with 95% ethanol and twice with water under reflux at 60°C. Two parts of extracts were mixed and placed in a freeze dryer until a powder was generated to afford the total extract (EIB) and stored in the refrigerator before use.

### 2.3. Cell Culture

The SMMC-7721, A375, U87-MG, MDA-MB-231, and SK-OV-3 cell lines were provided by Procell Life Science & Technology Co., Ltd. Cells were cultured in DMEM supplemented with 10% FBS in a 37°C with 5% CO_2_ incubator chamber.

### 2.4. MTT Assay

Cell viability was evaluated by the MTT assay. Cells were seeded into 96-well plates and treated with different concentrations of EIB (10, 20, 40, 80, 160, 320, and 640 *μ*g/mL). After 48 h, 20 *μ*L of MTT (5 mg/mL) reagent was added, and the plates were incubated for 4 h in the incubator chamber. Then, DMSO was added, and the absorbance was measured at 570 nm on a microplate reader (BioTek, America). The IC50 values were calculated to evaluate the toxic effect of EIB on cancer cells.

### 2.5. Cell Cycle Analysis

Cells were treated with EIB for 48 h, collected, washed twice with PBS, and fixed at 4°C with 75% frozen ethanol overnight. Then, cells were washed with PBS and were resuspended in 500 *μ*L. We added 20 *μ*L RNase A solution to 37°C water bath for 30 min. Then, 400 *μ*L PI dye solution was added, gently mixed, and incubated at 4°C for 1 h in the dark. Cell cycle analysis was performed with a FACSCalibur flow cytometer (BD Biosciences, USA) and analyzed by FlowJo 7.

### 2.6. Hoechst 33258 Staining

Cells were seeded into 24-well plates and treated with EIB at 37°C for 48 h. A total of 1 mL of 4% paraformaldehyde was added to each well for 10 min of fixation and washed twice with PBS. Hoechst staining solution was added and stained for 5 min. Cell apoptosis was observed under a fluorescence microscope. Three fields of each group were randomly selected for observation.

### 2.7. Annexin V-FITC/PI Double Staining Assay

The apoptosis detection kit was employed to determine the apoptosis by EIB on SMMC-7721 cells. After being treated with EIB, cells were harvested and washed twice with PBS and suspended in 400 *μ*L 1× Annexin V binding solution. We added 5 *μ*L Annexin V-FITC solution to the cell suspension, and the cells were mixed gently and incubated on ice for 15 min in the dark. Then, 10 *μ*L of PI staining solution was added, mixed gently, and incubated on ice for 5 min in the dark. Cells were analyzed for fluorescence with a flow cytometer.

### 2.8. Mitochondrial Membrane Potential Assay

Cells were treated with EIB in a 6-well plate. After 48 h, cells were collected and stained using JC-1 according to the manufacturer's protocol. The cells were resuspended in 0.5 mL DMEM, 0.5 mL of JC-1 staining solution was added, and the mixture was inverted several times and incubated at 37°C for 20 min. After that, they were washed twice with JC-1 staining buffer, resuspended in an appropriate amount of JC-1 staining buffer, and analyzed by flow cytometry.

### 2.9. Western Blot

After incubation with EIB for 48 h, cells were collected, rinsed twice with cold PBS, and lysed by RIPA buffer containing 1 mM PMSF on ice for 30 min, followed by 12,000 g for 5 min at 4°C. Protein lysate was separated by 8%, 10%, and 12% SDS-PAGE electrophoresis and then transferred to a PVDF membrane, and next, membranes are incubated with specific primary and secondary antibodies.

### 2.10. Mouse Xenograft Assay

The animal experiment was approved by the Animal Care and Use Committee of Anhui Medical University and followed the Chinese Guideline of Welfare and Ethics for Laboratory Animals. Thirty male 4-week-old BALB/c nude mice were purchased from the Model Animal Research Center of Nanjing University. After a quarantine period of one week, SMMC-7721 cells (4 × 10^6^) were suspended in 0.2 mL PBS and injected subcutaneously into the right flank of each mouse. When the tumor volumes were grown to 50–100 mm^3^, the mice were divided into five groups on average as follows: control, DDP (10 mg/kg/2d), EIB-300 (300 mg/kg/d), EIB-600 (600 mg/kg/d), and EIB-1200 (600 mg/kg/d), and the drug treatment began. When the tumor volumes of the control reached 1200 mm^3^ (approximately 1 g), the drug was stopped to sacrifice the mice. Mice weight and tumor sizes were monitored every 2 days, and tumor volume was calculated according to the formula *V*=(width)^2^ × length/2. At the end of the treatment, animals were sacrificed; tumors were taken out and weighed.

### 2.11. H&E and Immunohistochemistry Staining and TUNEL Assay

Tumor tissues were immersed in 4% polyoxymethylene, embedded in paraffin, and sectioned to a thickness. Part of them were stained by H&E, and the histopathological changes were observed under a light microscope. Immunohistochemical staining of tumor tissues was performed with antibodies against LC3. The TUNEL assay was performed according to the manufacturer's instructions by using the TUNEL Apoptosis Assay Kit (Beyotime, China), and the nucleus was stained with DAPI.

### 2.12. Statistical Analysis

Statistical analysis was performed using a one-way ANOVA with GraphPad Prism 6 and SPSS 16.0. *P* ≤ 0.05 was considered to indicate a statistically significant difference. All data are expressed in mean ± standard deviation (SD) for at least three separate experiments.

## 3. Results

### 3.1. EIB Significantly Decreased the Cell Viability of SMMC-7721

**Figure 1:**
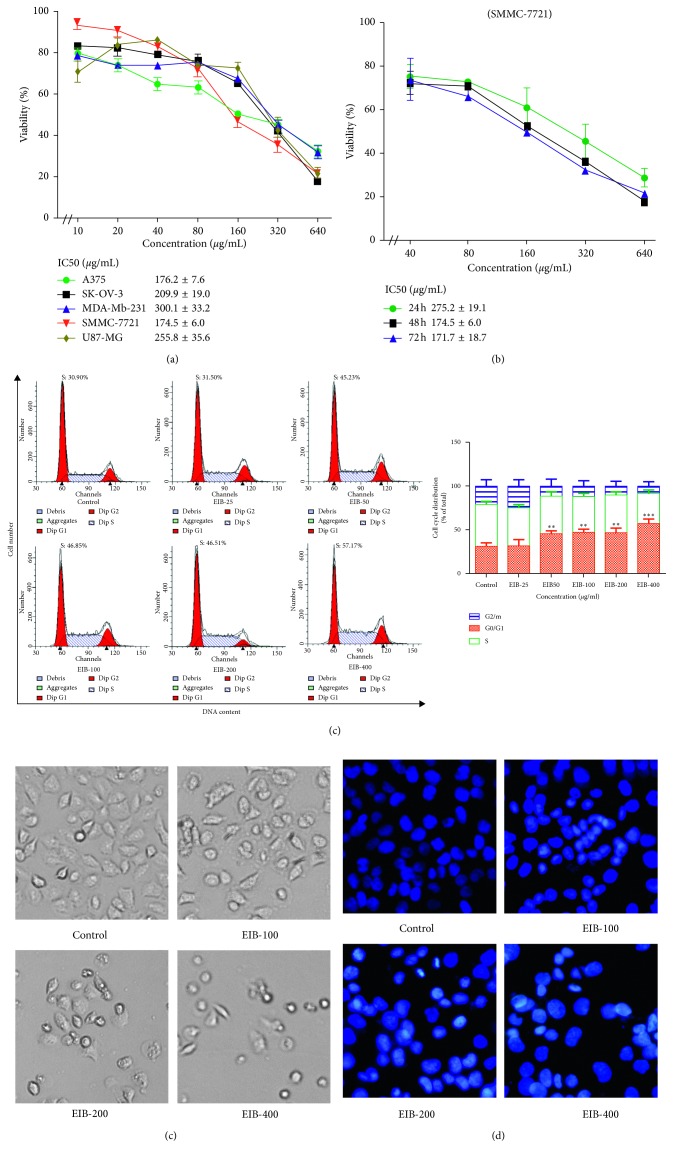
EIB inhibited the growth of SMMC-7721 cells. (a) Cell viability of A375, SK-OV-3, MDA-MB-231, SMMC-7721, and U87-MG cells analyzed by MTT. (b) MTT assay of SMMC-7721 for 24 h, 48 h, and 72 h. (c) Effect of EPI on cell cycle phase distribution. Cell cycle analyzed by flow cytometry and proportions of the cycle phase. ^*∗∗*^*P* < 0.01 and ^*∗∗∗*^*P* < 0.001 versus control. (d) Morphological changes of SMMC-7721 cells treated with EIB for 48 h. (e) Hoechst staining of SMMC-7721 cells detected by a fluorescent microscope after treating with EIB.

The cytotoxicity of EIB against cancer cells was evaluated by the MTT assay. As shown in Figure 1(a), after 48 h treatment, EIB showed an inhibitory effect on the cell proliferation of A375, SK-OV-3, MDA-MB-231, SMMC-7721, and U87-MG. Among them, EIB displayed the most cytotoxic activity against the liver cancer cell line SMMC-7721. Moreover, EIB exhibited a time-dependent inhibitory effect on the cell viability of SMMC-7721 (Figure 1(b)). Thus, a follow-up mechanistic investigation was conducted on this cancer cell line.

### 3.2. EIB Arrested the SMMC-7721 Cell Cycle at the S Phase

The cell cycle distribution of SMMC-7721 was analyzed by flow cytometry. As shown in Figure 1(c), there was no change for SMMC-7721 cells in the S phase at 25 *μ*g/mL (31.50%) after treatment with EIB for 48 h, but significant increase of cells at the S phase was observed from 50 *μ*g/mL (46.7% at 50 *μ*g/mL and 57.2% at 400 *μ*g/mL), compared with 30.9% for the control group. These data suggested that EIB blocked the SMMC-7721 cell cycle at the S phase.

### 3.3. EIB Induced the Morphological Changes of SMMC-7721 Cells

As shown in Figure 1(d), EIB-treated groups showed a significant reduction in the number of cancer cells compared with the control group, as well as the morphological changes of shrinkage, pyknosis, and the formation of floating cells. The Hoechst staining assay presented the typical apoptotic morphological changes including cytoplasmic shrinkage and chromatin condensation in SMMC-7721 cells compared with the control (Figure 1(e)).

### 3.4. EIB Induced Cell Apoptosis in SMMC-7721 Cells

**Figure 2:**
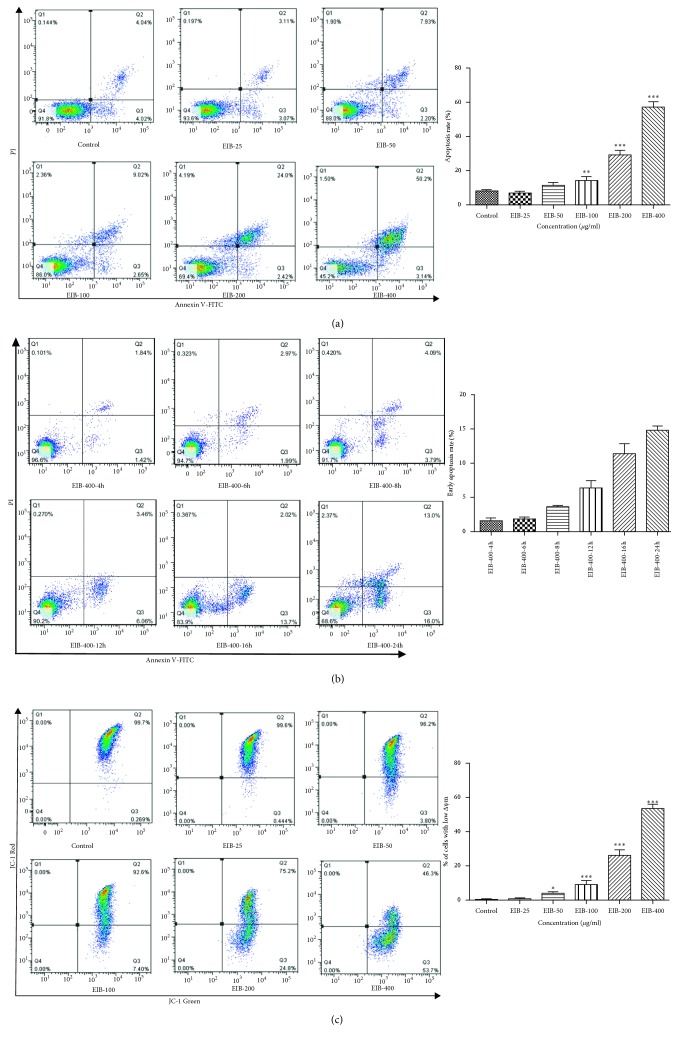
EIB induced apoptosis in SMMC-7721 cells. (a) Cell apoptosis was measured by the FITC-Annexin V/PI staining and flow cytometry in different concentrations. (b) Early apoptosis at 400 *μ*g/mL was measured by the FITC-Annexin V/PI staining and flow cytometry in different times. (c) EIB induced the collapse of mitochondrial membrane potential of SMMC-7721 cells. ^*∗*^*P* < 0.05, ^*∗∗*^*P* < 0.01, and ^*∗∗∗*^*P* < 0.001 versus control.

The percentage of cells undergoing apoptosis was determined by dual staining with Annexin V-FITC/PI by using a flow cytometer. As shown in Figure 2(a), the percentage of apoptotic cells for SMMC-7721 was 8.1% in the control group. After 48 h of exposure to 25, 50, 100, 200, and 400 *μ*g/mL EIB, the percentages of apoptotic cells for SMMC-7721 were 6.1%, 10.1%, 11.7%, 26.2%, and 53.3%, respectively. The data indicated that the apoptotic effect on SMMC-7721 cells induced by EIB was dependent on concentration. As shown in Figure 2(b), the percentages of early apoptotic cells for SMMC-7721 were, respectively, 1.34%, 1.76%, 3.55%, 5.47%, 13.0%, and 15.1% at 4, 6, 8, 12, 16, and 24 hours using the 400 *μ*g/ml dose.

### 3.5. EIB Decreased Mitochondrial Membrane Potential in SMMC-7721 Cells

Decreased mitochondrial membrane potential is a landmark event in the early stage of apoptosis. With JC-1 as a fluorescent probe, the decrease in cell membrane potential can be easily detected by the transition of JC-1 from red fluorescence to green fluorescence. As shown in Figure 2(c), after 48 h of EIB administration, the percentage of SMMC-7721 cells with reduced MMP increased significantly in a concentration-dependent manner.

### 3.6. EIB Regulated the Levels of Apoptosis-Related Proteins in SMMC-7721 Cells

**Figure 3:**
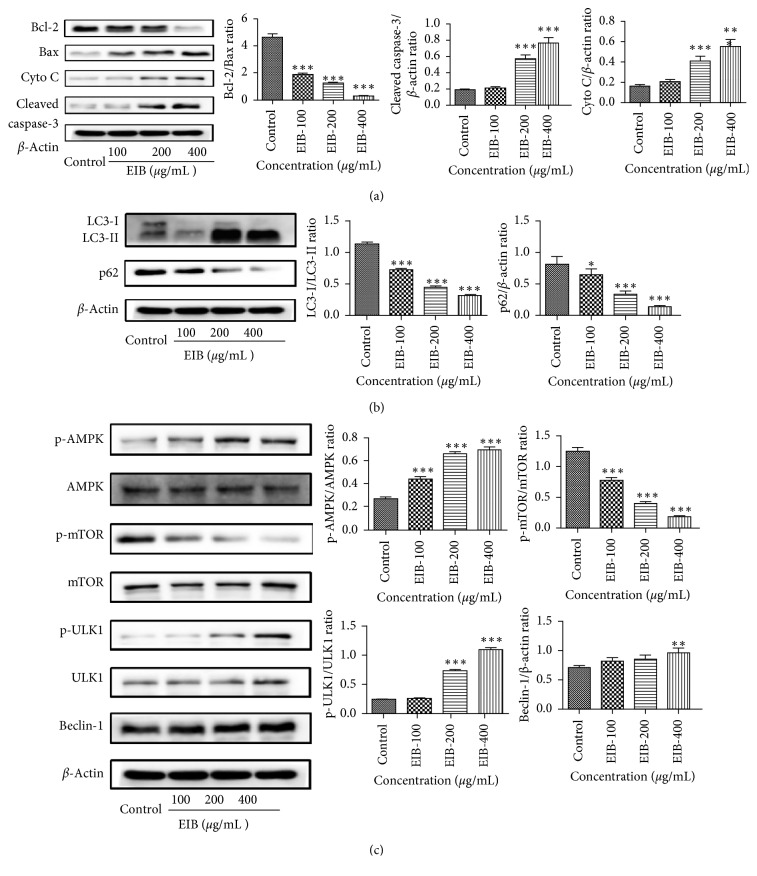
Western blot analysis was carried out to analyze the expression of apoptosis-related and autophagy-related proteins in SMMC-7721 cells. (a) EIB influenced the expressions of apoptosis-related protein, including Bcl-2, Bax, cytochrome c, and cleaved caspase-3. (b) EIB influenced the expressions of autophagy-related protein, including LC3-I, LC3-II, and p62. (c) EIB increased AMPK and ULK1 phosphorylation and reduced mTOR phosphorylation. EIB also increased the expression of Beclin-1. ^*∗*^*P* < 0.05, ^*∗∗*^*P* < 0.01, and ^*∗∗∗*^*P* < 0.001 versus control.

The proapoptotic and antiapoptotic members of the Bcl-2 family, such as Bcl-2 and Bax, regulate mitochondrial membrane integrity. Bcl-2 prevents the apoptotic process by interacting with Bax, blocking the release of cytochrome c from mitochondria into cytosol. As shown in Figure 3(a), the EIB treatment significantly increased the expression of Bax, cytochrome c, and cleaved caspase-3 in a concentration-dependent manner, while the Bcl-2 decreased remarkably. These results suggested that the induction of apoptotic cell death by EIB occurred.

### 3.7. Effect of EIB on the Autophagy-Related Proteins

LC3 and p62 are the marker proteins for autophagy. As shown in Figure 3(b), the ratio of LC3-I/LC3-II decreased in a dose-dependent manner after EIB was treated for 48 h, indicating that LC3-I was converted to LC3-II. In addition, the expression of p62 was also reduced. These results suggested that EIB induced autophagy in SMMC-7721 cells.

### 3.8. EIB Regulates the AMPK/mTOR/ULK1 Signaling Pathway in SMMC-7721 Cells

The rapamycin target protein (mTOR) is a key regulator of autophagy in mammalian cells and acts as a negative activator mediated by AMPK. As shown in our experiments, EIB increased protein levels of p-AMPK, p-ULK1, and Beclin-1, whereas decreased mTOR in a dose-dependent manner in SMMC-7721 (Figure 3(c)). These results indicated EIB induced SMMC-7721 cell autophagy via the AMPK/mTOR/ULK1 signaling pathway.

### 3.9. Treatment with EIB Significantly Inhibited Tumor Growth *In Vivo*

**Figure 4:**
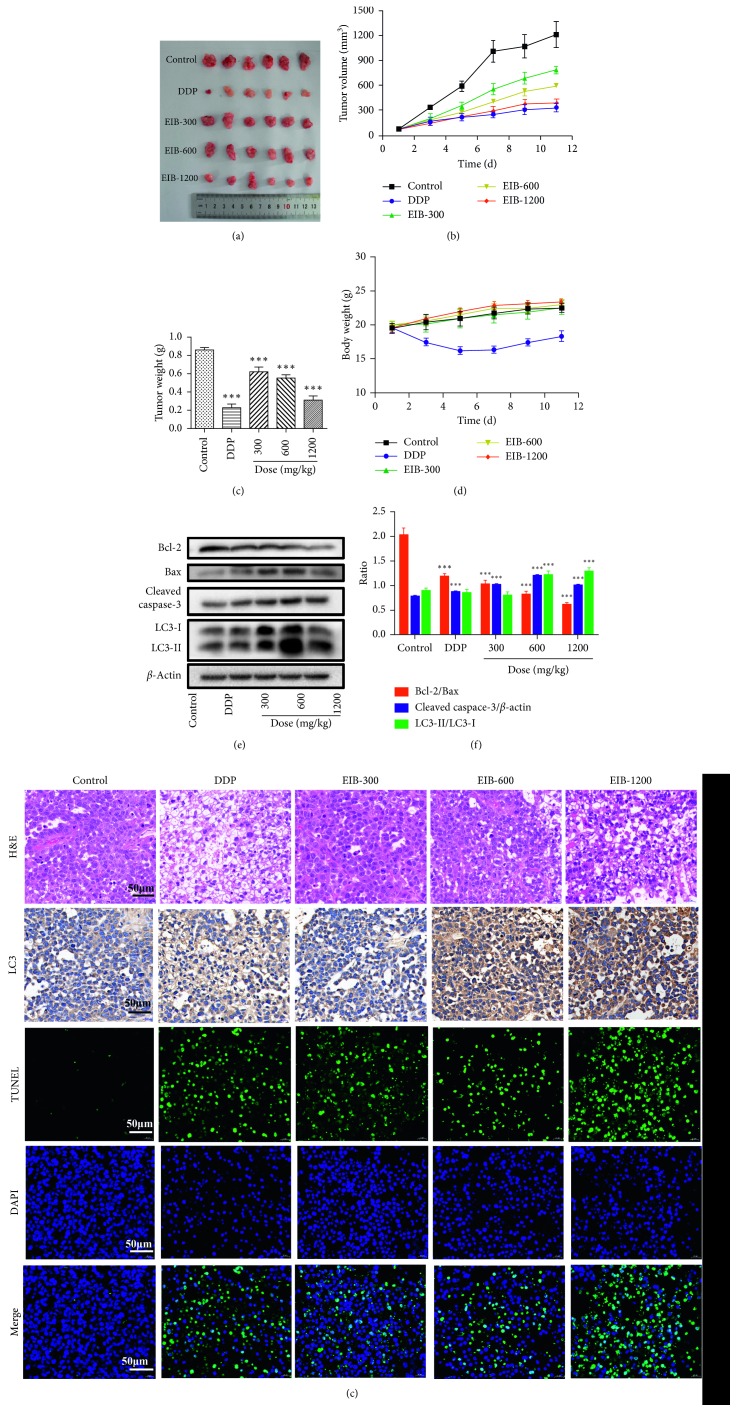
EIB inhibited the SMMC-7721 tumor growth in xenograft nude mice and mediated the expression of related proteins in tumor tissue. (a) EIB decreased the tumor size after 12 days of treatment, *n*=6. (b) EIB inhibited the tumor volume during the treatment. (c) EIB inhibited the tumor weight gain. (d) The body weight in each group. (e) EIB mediated the expression of apoptosis and autophagy-related proteins in tumor tissue in western analysis, *n*=3. (f) Densitometric quantification of the ration of Bcl-2/Bax, cleaved caspase-3/*β*-actin, and LC3-II/LC3-I. (g) Tumor sections stained with H&E for the histological examination, with TUNEL for the apoptosis analyses, and immunohistochemical analysis of the LC3 for the autophagy analyses. ^*∗∗∗*^*P* < 0.001 versus control.

Male BALB/c nude mice with SMMC-7721 cells of xenograft tumors were treated with vehicle, DDP, and three concentrations of EIB for 12 days. When tumors were taken out (Figure 4(a)), it was obvious that the tumors in the DDP and EIB treatment groups were smaller than those in the control group, and the inhibition was even more remarkable in the DDP and EIB-1200 treatment groups (Figures 4(b) and 4(c)). The body weights of EIB were higher than DDP (Figure 4(d)). The results of western blot showed that the expression levels of Bcl-2/Bax decreased and cleaved caspase-3 increased significantly versus the control. However, the expression levels of LC3-II/LC3-I in the groups of EIB-600 and EIB-1200 increased significantly. The results further validated that EIB could lead to SMMC-7721 cell death by apoptosis and autophagy (Figures 4(e) and 4(f)).

As shown in the first panel of Figure 4(g), H&E staining of tumor sections indicated that there were more survival cancer cells in tumors treated with the vehicle. In the group of control, the tumor tissue structure was clear, the cells were arranged neatly and closely, and there was nuclear fission, which shows a rapid growth state. While the cells in the group of DDP and EIB-1200 were significantly reduced, the cell arrangement was sparse and the volume was also reduced, and the nucleus was pyknotic, which was more obvious with the increase of the EIB dose. Then, induction of autophagy by EIB was investigated using IHC staining with LC3. As shown in the second panel of Figure 4(g), saline, DDP, and EIB-300 had few effects on the expression of LC3. However, the expression of LC3 was obviously increased by EIB-600 and EIB-1200, suggesting EIB could effectively induce autophagy of SMMC-7721 cells and achieved better therapeutic effect. Moreover, the images in the TUNEL assay presented a great quantity of apoptotic cells in the tumor tissues of the DDP and EIB group (the third to fifth panels of Figure 4(g)).

## 4. Discussion

HCC is the fourth common malignant tumor and the third-leading cause of cancer death in China [2, 3]. Unfortunately, current therapeutic options for HCC are far from satisfactory due to various adverse effects and poor prognosis. Therefore, the development of effective and safe therapeutic agents is urgently needed. Growing studies have suggested that some medicinal fungi had the effects to prevent cancers [18]. In traditional Chinese medicine, *I. baumii* is recorded “Sanghuang,” which is documented as a rare natural medicine with a variety of health benefits including cancer treatment [14]. In the present study, the MTT assay showed that EIB could inhibit the viability of five human cancer lines of A375, SK-OV-3, MDA-MB-231, SMMC-7721, and U87-MG *in vitro*, especially against the SMMC-7721.

The cell cycle consists of a series of highly ordered sequential phases that lead to cell division [19]. Uncontrolled cell proliferation is the hallmark of cancers, and cancer cells have typically acquired mutations in genes that directly regulate their cell cycle. Cell cycle arrest is considered as an effective strategy for controlling cancer [20]. In the present study, cell cycle analysis showed that EIB blocked SMMC-7721 cancer cells at the S phase.

Apoptosis and autophagy are two classical forms of cell death with distinct morphological features by activating specific signaling pathways [9]. Apoptotic cells display morphological characteristics, including cell shrinkage, chromosome condensation, nuclear fragmentation, plasma membrane blebbing, and the formation of apoptotic bodies [10]. These morphological changes and disappearance of cells could be the indication of cell death and a reliable basis for the judgment of cells undergoing apoptosis [21]. In this study, the Hoechst assay and the Annexin V-FITC/PI staining assay were performed to detect whether EIB induced apoptosis. The significant changes in cell morphology and increasing rates in apoptosis suggested that EIB may induce apoptosis in SMMC-7721 cells.

The Bcl-2 family of proteins controls the apoptosis pathway by regulating the permeability of the mitochondrial outer membrane [22], releases cytochrome c from the mitochondria into the cytoplasm to form the apoptosome together with Apaf-1 and procaspase-9 [23], and induces caspase-9 and caspase-3 activation, which activates effector caspases to trigger a cascade of proteolytic events, causes the cell phosphatidylserine translocation and DNA fragmentation, and leads to apoptosis [10, 24]. The results showed that EIB upregulated the expression of Bax and downregulated Bcl-2 in SMMC-7721 cells. EIB resulted in the mitochondria outer membrane permeabilization indicated by the loss of mitochondrial membrane potential. Moreover, the two key molecules, cytochrome c and caspase-3, were significantly upregulated after the EIB treatment. These results suggested that EIB induced apoptosis in SMMC-7721 cells.

Autophagy is a self-degradation cellular process that functions in cell survival/death under certain circumstances [25, 26]. Autophagic cell death is an important mechanism for cancer therapy [27, 28]. Some natural medicines are reported to cause tumor cell death through autophagy [29]. LC3, a structural component in the formation of autophagosomes, is a molecular marker for the promotion of autophagy. During autophagy, LC3-I is modified to LC3-II and bound to the autophagosome membrane. The synthesis and membrane translocation of LC3-II are considered a hallmark of autophagosome biogenesis. p62, another related marker of autophagy, is a critical autophagy substrate that is incorporated into autophagosomes through its interaction with LC3 and is gradually degraded by autophagy [30]. Western blot analysis showed that the expression level of LC3-II in SMMC-7721 cells was significantly increased after the EIB treatment, whereas p62 was decreased. Collectively, these suggested that EIB induced autophagy in SMMC-7721 cells.

Autophagy is regulated by a complex signaling network, most of which feed into the AMPK/mTOR/ULK pathway [31]. ULK is the initiator of autophagy. The master signal that triggers autophagy comes from the ULK1 complex [32]. Two upstream kinases, mTOR and AMPK, control ULK1 activation, the former acting as a repressor and the latter acting as an activator. Our data showed that EIB suppressed the level of p-mTOR and increased p-AMPK and p-ULK1 levels in SMMC-7721 cells, indicating that the AMPK/mTOR/ULK1 signaling pathway was involved in autophagy induced by EIB.

As EIB could availably suppress the proliferation and induce cells death *in vitro*, we conducted a study *in vivo* to determine its efficacy in inhibiting the tumor growth in xenograft nude mice. In our study, compared with the control, the tumor volume growth rate in the drug-administered group was significantly slower. At a dose of EIB-1200, the therapy was more tolerated, while the DDP was more effective. Compared with DDP, the animals treated with EIB suffered fewer side effects, obviously that the body weights were higher, the figure was as follows. The cell cycle analysis suggested that EIB blocked the SMMC-7721 cell cycle at the S phase. It may be a similar mechanism with DDP. However, there were many additional benefits of EIB; it was reported that *I. baumii* has the functions of immune regulation [14] and anti-inflammatory [33] and antioxidant [34] activity, which are related to anticancer activity, while the adverse reactions of cisplatin and some other drugs are very large. Therefore, EIB might be a complementary and alternative medicine.

The H&E staining results confirmed the inhibition of EIB on the transplanted tumor of SMMC-7721 nude mice from pathological morphology. The TUNEL staining showed that EIB promoted apoptosis in a dose-dependent manner *in vivo*; IHC staining showed that EIB induced autophagy, and western blot confirmed that at the molecular level.

Many studies demonstrated the multicomponent, multitarget, and multichannel characteristics of TCM [35–38]. It acquires special treatment efficacy by acting on the biological network of body systems. EIB exerted the antitumor potential through various paths. We have demonstrated that EIB inhibited cell proliferation and arrested cell cycle at the S phase. The Bcl-2 family, caspase family, and AMPK/mTOR/ULK1 pathway were involved in the cell death.

In general, the therapeutic effects of herbal medicines are based on mixtures of different compounds, which often act in concert to exert all their beneficial effects. It was reported that polysaccharide (PPB-2) extracted and purified from the fruiting body of *I. baumii* had hypoglycemic and hepatoprotective activities [39]. Phelligridin D and other compounds had anti-inflammatory activity [40, 41]. However, little publications reported that compounds extracted from *I. baumii* had antitumor activities. Therefore, it is of great significance to study the material basis and biological function of compounds from *I. baumii* for anticancer in the future.

## 5. Conclusions

In summary, our investigations demonstrated that the extract of *Inonotus baumii* inhibited the proliferation in SMMC-7721 cells and induced cell death, in which apoptosis and autophagy might be involved. These findings provide a further understanding of the mechanism by which *I. baumii* acts as a potential agent for the prevention and treatment of cancers including HCC. Future research is needed to focus on identifying bioactive compounds that play a role in this medicinal fungus.

## Data Availability

The data used to support the findings of this study are available from the corresponding author upon request.
